# Cultured bovine granulosa cells rapidly lose important features of their identity and functionality but partially recover under long-term culture conditions

**DOI:** 10.1007/s00441-017-2571-6

**Published:** 2017-02-02

**Authors:** Vengala Rao Yenuganti, Jens Vanselow

**Affiliations:** Institute of Reproductive Biology, Leibniz Institute for Farm Animal Biology (FBN), Wilhelm-Stahl-Allee 2, 18196 Dummerstorf, Germany

**Keywords:** Cell plating, Cell identity, *FOXL2*, *SOX9*, *PTGS2*

## Abstract

Cell culture models are essential for the detailed study of molecular processes. We analyze the dynamics of changes in a culture model of bovine granulosa cells. The cells were cultured for up to 8 days and analyzed for steroid production and gene expression. According to the expression of the marker genes *CDH1*, *CDH2* and *VIM*, the cells maintained their mesenchymal character throughout the time of culture. In contrast, the levels of functionally important transcripts and of estradiol and progesterone production were rapidly down-regulated but showed a substantial up-regulation from day 4. *FOXL2*, a marker for granulosa cell identity, was also rapidly down-regulated after plating but completely recovered towards the end of culture. In contrast, expression of the Sertoli cell marker *SOX9* and the lesion/inflammation marker *PTGS2* increased during the first 2 days after plating but gradually decreased later on. We conclude that only long-term culture conditions (>4 days) allow the cells to recover from plating stress and to re-acquire characteristic granulosa cell features.

**Electronic supplementary material:**

The online version of this article (doi:10.1007/s00441-017-2571-6) contains supplementary material, which is available to authorized users.

## Introduction

Granulosa cells are essentially involved in the production of various endocrine- and paracrine-acting hormones such as inhibin, follistatin and estradiol (E2). In order to analyze molecular processes within granulosa cells under diverse pathophysiological conditions, the establishment of appropriate culture models is required. During the last two decades, several different culture systems have been described and used for detailed molecular studies. In the bovine, Gutierrez et al. (1997) established a serum-free follicle-stimulating hormone (FSH)-responsive E2-producing granulosa cell culture system. Moreover, later studies clearly showed that considerable E2 secretion and the expression of corresponding key transcripts by these cells could only be observed under serum-free culture conditions plus FSH and insulin-like growth factor-1 (IGF-1) stimulation (Hamel et al. 2005; Silva and Price 2000). In another study, serum supplementation was demonstrated to induce proliferation but reduced or abolished steroid production and the expression of functionally important genes (Baufeld and Vanselow 2013). To validate a culture model, the functionality and identity of the cells need to be assessed based on physiological and molecular characteristics. Granulosa cells are of mesenchymal origin and express *CDH2* (cadherin 2) and *VIM* (vimentin), whereas oocytes express the epithelial cell marker *CDH1* (cadherin 1; Mora et al. 2012). During follicular growth and differentiation, the proper regulation of genes, such as *CYP19A1* encoding cytochrome P450, family 19, subfamily A, polypeptide 1, *CCND2* encoding cyclin-D2, *FSHR* encoding follicle-stimulating hormone receptor and *LHCGR* encoding luteinizing hormone/choriogonadotropin receptor and of other genes that are important for granulosa cell function (Gonzalez-Robayna et al. 2000; Park et al. 2005; Law et al. 2013) is essential. Expression of *CYP19A1*, the key gene of estradiol production, is regulated by various factors such as *FOXL2* encoding forkhead box protein L2 and *NR5A2* encoding nuclear receptor subfamily 5, group A, member 2, together with FSH signaling (Sahmi et al. 2014). *FOXL2* has emerged as a key factor of ovarian biology. Granulosa cells maintain their identity by expressing *FOXL2* and repressing the Sertoli cell marker *SOX9* encoding SRY-box 9 (Georges et al. 2014; Uhlenhaut et al. 2009). Knockdown of *FOXL2* leads to the loss of granulosa cell identity and the gain of Sertoli cell properties (Uhlenhaut et al. 2009; Ottolenghi et al. 2007). Moreover, *FOXL2* regulates the expression of other functionally important genes such as *ESR2* encoding estrogen receptor 2 and *FST* encoding follistatin.

Until now, our knowledge about the dynamics of changes that are induced by the dissociation, plating and culture of granulosa cells has been limited. This knowledge is however an important pre-requisite for appropriately designing in vitro experiments with cultured granulosa cells. Therefore, during the present study, we analyzed the progressive changes in the physiological and molecular characteristics in an estrogen-active granulosa cell culture model. The production of the steroids E2 and P4 (progesterone) and the expression of marker genes for granulosa cell functionality and identity were analyzed over 8 days and compared with those of freshly isolated cells.

## Materials and methods

### Culture of granulosa cells

The chemicals and antibodies used are shown in the Electronic Supplementary Material (Materials and Methods S1).

Granulosa cells were cultured as previously described (Baufeld and Vanselow 2013; Yenuganti et al. 2016). The cells were aspirated from small to medium-sized follicles (2–6 mm in diameter) from slaughterhouse material and plated on collagen-coated 24-well plates with 1.25 × 10^5^ viable cells (as determined by the trypan blue exclusion method) per well. This isolation procedure enabled nearly pure granulosa cells to be obtained with no contaminating theca cells (Nimz et al. 2009). Cells were grown for up to 8 days in serum-free α-MEM containing L-glutamine (2 mM), sodium bicarbonate (0.084%), bovine serum albumin (BSA; 0.1%), HEPES (20 mM), sodium selenite (4 ng/ml), transferrin (5 μg/ml), insulin (10 ng/ml), non-essential amino acids (1 mM), penicillin (100 IU) and streptomycin (0.1 mg/ml) with FSH (20 ng/ml) and IGF-1 (25 ng/ml) stimulation and androstenedione (2 μM) supplementation at 37 °C in a 5% CO_2_ atmosphere. Media containing all supplements were replaced every other day.

### RNA isolation, cDNA synthesis and real-time reverse transcription polymerase chain reaction

Fore RNA isolation, attached cells were washed once with phosphate-buffered saline (PBS) before lysis. Total RNA was isolated by the Nucleo Spin RNA II Kit (Macherey-Nagel, Düren, Germany) and quantified with a NanoDrop1000 Spectrophotometer (Thermo Scientific, Bonn, Germany). The cDNA was prepared by using the SensiFAST cDNA Synthesis Kit from 200 ng RNA (Bioline, Luckenwalde, Germany).

Transcript abundance levels were measured by real-time reverse transcription polymerase chain reaction (qPCR) and calculated relative to *TBP* (TATA-binding protein) housekeeping transcripts (Baddela et al. 2014) for normalization. qPCR was performed with SensiFast SYBR No-ROX (Bioline) and gene-specific primers (see Electronic Supplementary Material, Table S1) in a Light Cycler 96 instrument (Roche, Mannheim, Germany) as described previously (Baddela et al. 2014; Yenuganti et al. 2016). Normalized qPCR values were then expressed as fold changes relative to the respective transcript abundance found in freshly isolated cells.

### Western blotting

After being washed twice with 500 μl PBS, the cells were scraped off from culture wells in 500 μl PBS, subsequently centrifuged at 135 relative centrifugal force (rcf) for 2 min, washed with PBS, collected in 500 μl PBS and centrifuged again at 135 rcf for 2 min. Cell pellets were re-suspended in 50 μl RIPA buffer and sonicated (LABSONIC M, Sartorius, Göttingen, Germany) at 0.5 cycles and an amplitude of 30% for 2 × 20 times with a few seconds break. The suspension was centrifuged at 18,400 rcf for 2 min and the protein concentration of the supernatants was measured by a Micro BCAProtein Assay Kit. Proteins were separated on 12.5% polyacrylamide gels (0.75 mm) by electrophoresis at 20 mA (stacking gel) and 30 mA (separating gel). The gels were electro-transferred to Immobilon-P Membrane for 60 min at 1 mA/cm^2^ in a Pierce fast semi-dry blotter apparatus (Dreieich, Germany). The membranes were then washed with TBST (TRIS-buffered saline and Tween 20) containing 0.1% Tween, blocked in a SNAP protein detection system (Millipore) with 30 ml blocking solution (0.5% milk powder with TBST), washed three times with 30 ml TBST and incubated with FOXL2 (1.5 μg/ml) and SOX9 (1:1000 dilution) antibodies in TBST with 5% BSA and incubated at 4 °C overnight. The membranes were then washed three times with 30 ml TBST each for 10 min, incubated with the secondary antibody in blocking solution (1:3000) for 90 min at room temperature and washed with 30 ml TBST (three times each) for 10 min. To detect the proteins, 5 ml Amershan ECL prime Western blotting detection reagent was added to the membranes, which were then incubated for 5 min and exposed to X-ray films. The films were developed for 1 min, subsequently washed for 30 s in water and fixed for 2 min. After a drying step, the images were captured by Raytest (Isotopenmeßgeräte, Staubenhardt, Germany).

### Quantification of E2 and P4

For the determination of steroids, conditioned media were removed from individual wells. Concentrations of E2 and P4 in conditioned culture media were quantified as described previously (Schneider and Brüssow 2006; Baufeld and Vanselow 2013; Yenuganti et al. 2016). Details of the assays are shown in the Electronic Supplementary Material (Materials and Methods S1). E2 and P4 values were expressed relative to the amounts of total RNA isolated from individual samples as a surrogate for the respective cell numbers.

### Statistical analysis

All experiments were carried out three times independently and all data were analyzed by one-way analysis of variance (ANOVA) following Tukey’s multiple comparison test by using GraphPad prism 5.0 software.

## Results

### Effects of plating on steroid hormone production

Hormone analysis showed that relative E2 and P4 values were low up to day 4 but that they strongly and significantly increased from day 6 (Table 1). The absolute E2 values in conditioned media after 8 days was 123.3 ± 5.7 ng/ml (mean ± SEM), which is similar to concentrations found in the follicular fluid of bovine dominant E2 active follicles (Nimz et al. 2009), whereas the respective P4 values (788.7 ± 38.9 ng/ml) were much higher than those found in vivo before the pre-ovulatory luteinizing hormone (LH) surge.

**Table 1:** Estradiol (*E2*) and progesterone (*P4*) concentrations relative to total RNA content in conditioned media from granulosa cells after various culture periods (different *superscripts* indicate significantly different means ± standard errors; *P* < 0.05; one-way ANOVA)

Hormone	1 day	2 days	4 days	6 days	8 days
E2/RNA	0.35 ± 0.08^a^	0.38 ± 0.10^a^	0.72 ± 0.05^a^	2.35 ± 0.59^b^	3.28 ± 0.23^b^
P4/RNA	4.99 ± 0.70^A^	5.03 ± 0.89^A^	8.69 ± 0.55^A,B^	13.00 ± 0.50^B^	23.21 ± 3.23^C^

### Effects of plating on abundance of functionally important transcripts

**Fig. 1:**
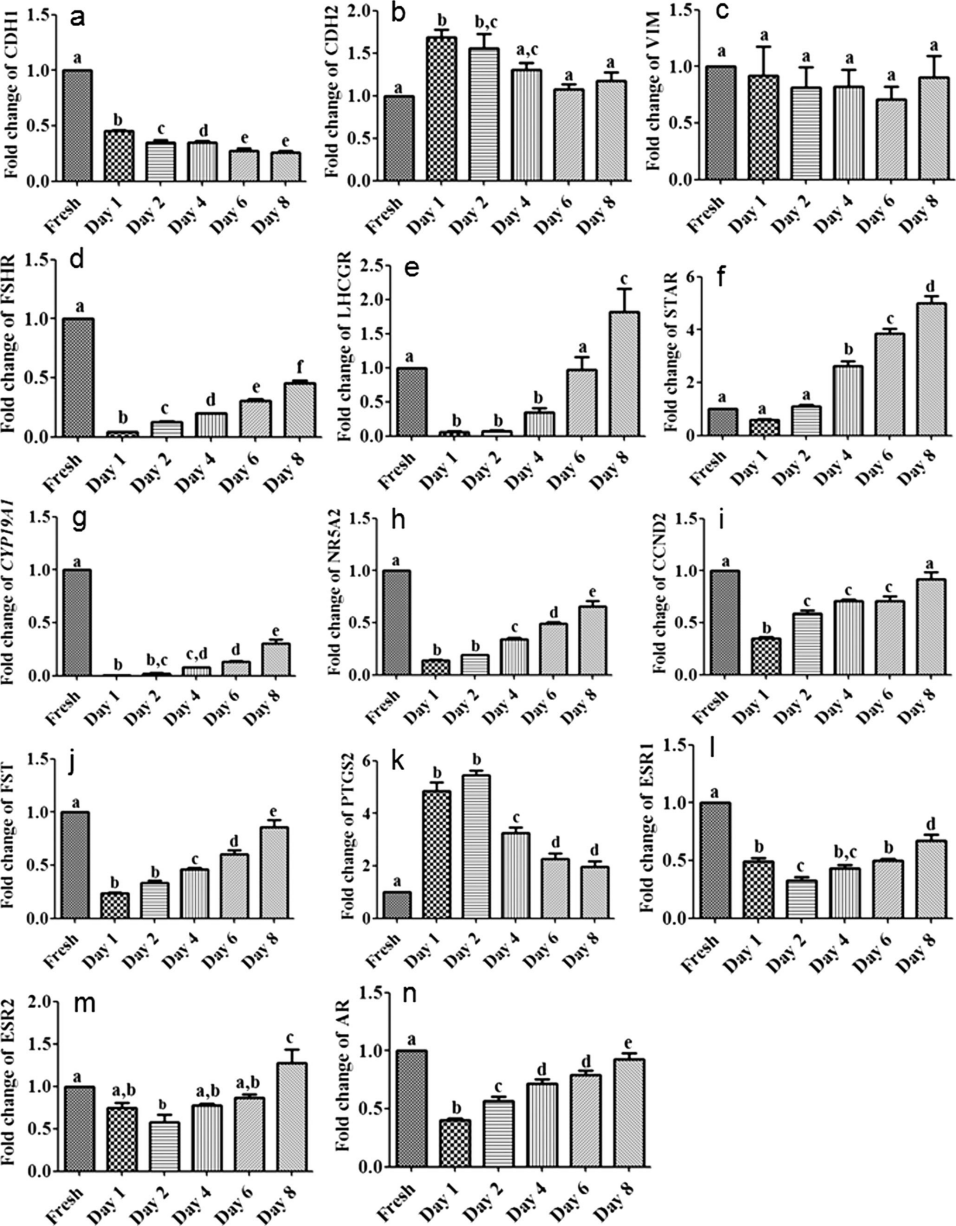
Abundance levels of functionally important transcripts in freshly isolated and cultured granulosa cells. Abundance levels of different transcripts (**a**–**n**) are shown in freshly isolated (*Fresh*) and plated granulosa cells after various culture periods (days 1 to 8). Different *letters* indicate significantly different changes (mean fold changes ± standard errors; *P* < 0.05; one-way analysis of variance [ANOVA] from three independent experiments) relative to freshly isolated cells

To elucidate whether plating induced a mesenchymal to epithelial transition, we analyzed the expression of mesenchymal (*CDH2* and *VIM*) and epithelial (*CDH1*) markers. *CDH1* was down-regulated in cultured cells as compared with the freshly isolated cells with the lowest levels from day 6. The expression of *CDH2* was up-regulated with the highest levels at day 1 and 2 but reduced levels similar to those found in freshly isolated cells were detected later on. In contrast, the levels of VIM were not regulated under culture conditions with almost similar levels in cultured and freshly isolated cells (Fig. 1a–c).

An appropriate response to gonadotropins is essential for the function of granulosa cells. Therefore, we comparatively measured the expression levels of *FSHR* and *LHGCR* in freshly isolated and in plated granulosa cells. Levels of *FSHR* transcripts rapidly decreased after plating and continuously increased from day 2 but did not reach the levels found in freshly isolated cells. Moreover, the expression of *LHCGR* transcripts rapidly decreased after plating and started to increase later on. However, in contrast to *FSHR*, the abundance of *LHCGR* transcripts reached and even exceeded the level of that in freshly isolated cells after 6 days (Fig. 1d, e).

As a paradigm for steroid hormone production, we analyzed the expression of steroidogenic acute regulatory protein (*STAR*) and of *CYP19A1* (Fig. 1f, g). In addition, we analysed the expression of the nuclear receptor *NR5A2*, which regulates several functionally important genes in granulosa cells, such as *CCND2*, which is involved in the regulation of granulosa cell proliferation (Robker and Richards 1998), *FST*, which is involved in feedback regulation between brain and ovary and *PTGS2*, which is essential for ovulation. *CYP19A1*, *NR5A2*, *CCND2* and *FST* were rapidly down-regulated, with *CYP19A1* decreasing to nearly undetectable levels after cell plating (Fig. 1g–j). Subsequently, a continuous up-regulation of these transcripts was observed from days 2 or 4. However, in particular, *CYP19A1* transcripts but also *NR5A2* and *FST* transcripts, did not reach the original levels found in freshly isolated granulosa cells. In contrast, *STAR* and *PTGS2* transcripts (Fig. 1f, k) showed higher expression in cultured granulosa cells. However, whereas the abundance of *STAR* transcripts continuously increased from day 4, the expression of *PTGS2* was rapidly up-regulated after plating but subsequently declined. Estrogens and androgens exert their action on granulosa cells via various receptors, namely estrogen receptor 1 (*ESR1*), estrogen receptor 2 (*ESR2*) and androgen receptor (*AR*). The analysis of the respective transcripts showed an initial decrease and a continuous increase after plating (Fig. 1l–n).

### Expression of marker transcripts and proteins of granulosa and sertoli cell identity

**Fig. 2:**
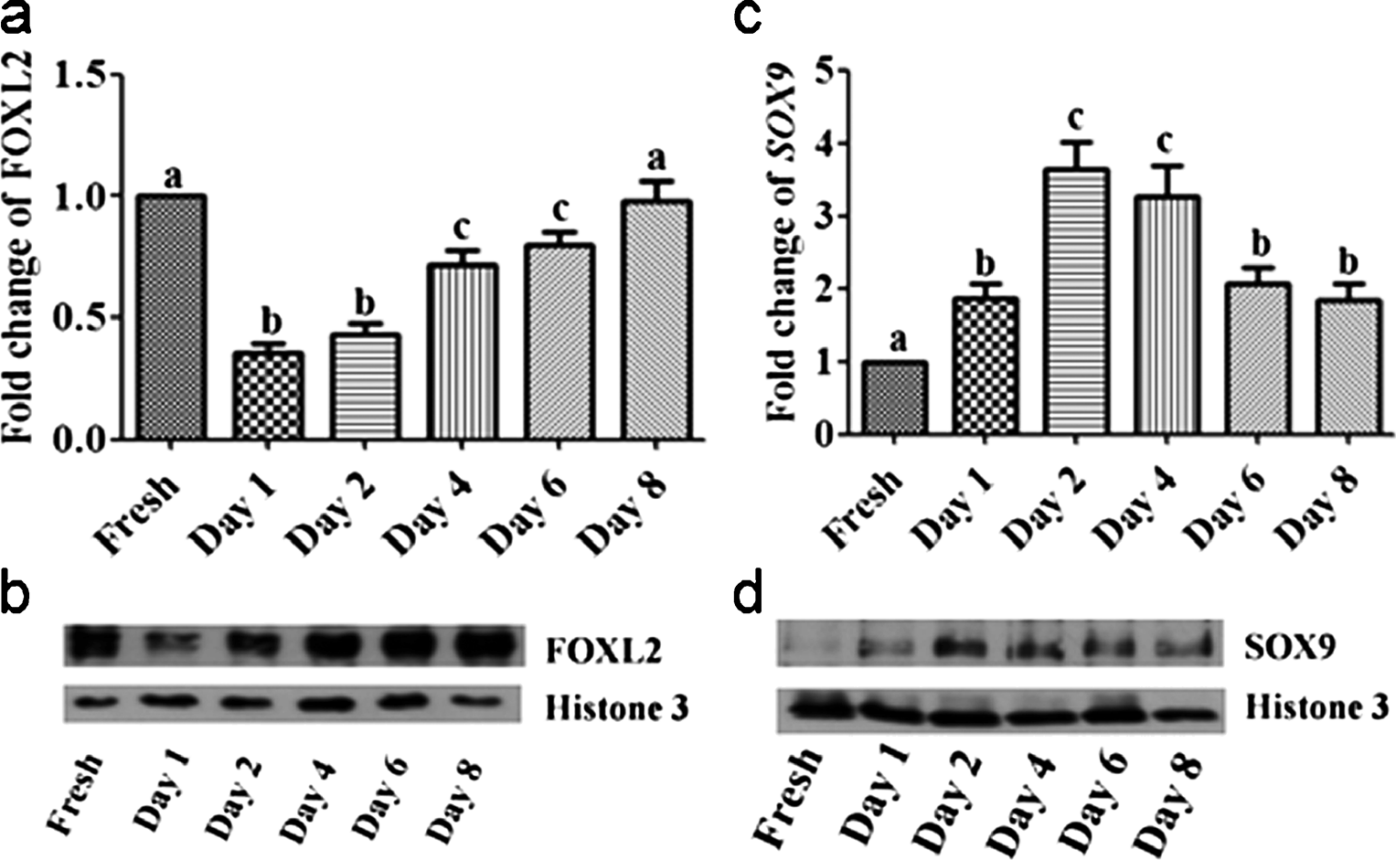
Transcript and protein abundance of *FOXL2* and *SOX9* in freshly isolated and cultured granulosa cells. Transcript and protein abundance of *FOXL2* (**a**, **b**) and of the Sertoli cell marker *SOX9* (**c**, **d**) as determined by RT-qPCR (**a**, **c**) and immunoblotting (**b**, **d**; representative blots). Different *letters* indicate significant differences of fold changes (mean fold change ± standard error; *P* < 0.05; one-way ANOVA from three independent experiments) relative to freshly isolated cells

Because of the very low initial *CYP19A1* expression and E2 production in cultured cells as compared with those in freshly isolated samples, we analyzed the mRNA and protein expression of markers of granulosa and sertoli cell identity, namely *FOXL2* and *SOX9*, respectively. Interestingly, *FOXL2* expression was reduced but that of *SOX9* was increased after plating. However, whereas the expression of *FOXL2* rapidly decreased and slowly recovered, thus reaching similar levels as those observed in freshly isolated granulosa cells, the levels of *SOX9* were low in freshly isolated granulosa cells but increased after plating and decreased again towards the end of culture (Fig. 2).

## Discussion

To our knowledge, the present study describes, for the first time, the dynamics of changes that are induced by the dissociation, plating and culture of granulosa cells based not only on physiological characteristics but also on numerous marker genes that are important for the identity, structure and functionality of granulosa cell. In contrast to previous studies, our presented set of data also allows a direct comparison of the in vitro data with the data derived from original in vivo material, i.e., freshly isolated cells.

The present results showed that some plated granulosa cells strongly changed their expression levels of some marker genes. However, the decreased expression of the epithelial marker *CDH1* but the constant or even increased expression of the mesenchymal markers *VIM* and *CDH2*, respectively, clearly suggest that aspirated granulosa cells maintain their mesenchymal character under the present culture conditions and do not shift towards an epithelial phenotype.

In contrast, our data clearly indicate that plating severely and rapidly compromises granulosa cell functionality, in particular during the first few days in culture. The levels of gonadotropin receptor transcripts, namely *FSHR* and *LHCGR* and in particular those of *CYP19A1* immediately and strongly decreased, with those of *CYP19A1* being reduced to nearly negligible levels during the first 2 days in culture. Later on, the cells continuously started to re-express these genes but only in the case of *LHCGR* did the transcript levels reach and even exceed those found in freshly isolated granulosa cells. The dynamics of *CYP19A1* expression is also clearly in agreement with E2 production showing an increase from very low levels directly after plating to its highest levels at the end of culture. Moreover, the levels of P4 gradually increased with time in culture. However, the final levels were much higher than the corresponding E2 levels. This is in concordance with the remarkably increased expression of *STAR*, which is involved in the first step of steroid hormone biosynthesis. On the other hand, this also suggests that the cells display some properties of luteinized granulosa cells, which are characterized by a huge increase of P4 production. However, the simultaneous ability of the cells to produce E2 at physiological levels clearly contradicts the notion that the cells have passed the folliculo-luteal transition phase but instead suggests that they might be arrested at an intermediate stage even after long-term culture.

In bovine granulosa cells, the activation of *CYP19A1* expression has been shown to require FSH stimulation and the presence of the transcription factors *FOXL2* and *NR5A2* (Sahmi et al. 2014). Interestingly, the expression of *FSHR*, *NR5A2* and *FOXL2* was also rapidly down-regulated after plating, thus suggesting that this might in turn cause the down-regulation of *CYP19A1* transcripts and of E2 production. In addition, the levels of *CCND2* and *LHCGR* that are also regulated by FSH signaling were transiently down-regulated directly after plating.

Previous reports have shown that *FOXL2* is important for maintaining granulosa cell identity, whereas *SOX9* is described as a Sertoli cell marker (Georges et al. 2014; Uhlenhaut et al. 2009). *FOXL2* expression was low in our culture system directly after plating, with the lowest levels at day 2 and gradually increased from day 4. At the same time, the expression of *SOX9* was stimulated after plating, thus reaching a maximum level at day 2, before it was subsequently down-regulated. This suggests that reduced levels of *FOXL2* expression after plating allow an increased expression of *SOX9* and thus might induce a transient loss of granulosa cell identity and a gain of Sertoli cell characteristics. In contrast, genes whose expression is usually stimulated by *FOXL2*, such as *FST* (Georges et al. 2014), were down-regulated.

Estradiol exerts its actions via the nuclear receptors, *ESR1* and *ESR2*. In the bovine, *ESR2* mRNA and protein are present in granulosa cells (Rosenfeld et al. 1999). Our data showed that *ESR2* was initially down-regulated after cell plating, with a minimum at day 2, before it was continuously up-regulated towards the end of culture. *ESR1* expression rapidly decreased in plated granulosa cells but, in contrast to *ESR2*, did never reach its original levels as observed in freshly isolated cells. The temporal expression pattern of *ESR2* paralleled the expression of *FOXL2* but not that of *ESR1*. Georges et al. (2014) showed that *ESR2* is essential for estradiol-mediated signaling in granulosa cells. These data suggest that the plating of bovine granulosa cells will, at least during the first few days in culture, reduce the E2 responsiveness of these cells. Moreover, the levels of *AR* were rapidly down-regulated after plating but subsequently recovered up to day 8. Sen and Hammes (2010) demonstrated that mice with granulosa-cell-specific *AR* knockout exhibit severe ovarian defects. This adds to our conclusion that granulosa cell functionality is severely compromised during the first few days after plating.

Follistatin is a glycoprotein that binds to activin and inhibits its activity (Shimasaki et al. 1989), thus acting as an inhibitor of FSH secretion from the pituitary (Robertson et al. 1987; Ueno et al. 1987). In addition to these endocrine actions, follistatin is known to be able to act as a paracrine or autocrine antagonist of activin within the bovine follicle. *FST* transcripts are highly up-regulated in large estrogen-active follicles (Glister et al. 2011). The immediate down-regulation of *FST* transcript abundance after plating and slow recovery under long-term culture conditions again suggests that bovine granulosa cells rapidly lose important molecular properties under culture conditions but can recover these properties several days later.

Interestingly, our results showed a rapid rise and subsequent decrease of *PTGS2* expression with levels near to those found in freshly isolated cells. In vivo *PTGS2* expression is considered as a reliable marker for approaching ovulation (Sirois 1994). *PTGS2* encodes COX-2, which is the key enzyme in prostaglandin biosynthesis. In addition to its role in ovulation, the expression of this enzyme is induced after lesions or inflammatory reactions. Accordingly, the rapid and transient up-regulation of *PTGS2* after plating can be interpreted as the result of a cellular stress reaction attributable to the dissociation and plating procedure. Its down-regulation several days later might therefore, in turn, indicate that the cells have largely recovered from the initial stress.

From our results, we conclude that, after being plated, granulosa cells rapidly but transiently, lose important features of their identity and functionality as indicated by the down-regulation of functionally important genes. Moreover, the transient up-regulation of the stress marker *PTGS2* and of the Sertoli cell marker *SOX9* supports this view. However, after long-term culture, those markers that are important for granulosa cell functionality gradually increase, whereas those that compromise granulosa cell function, such as the Sertoli cell and stress/inflammation markers, gradually decrease towards day 8. Accordingly, under the present culture conditions, the cells need at least 4 days to recover from the plating stress thus suggesting that granulosa cells cultured for only 4 days or less might not be an appropriate in vitro model to analyze granulosa cell function.

## Electronic supplementary material

Below is the link to the electronic supplementary material.

ESM 1 (DOCX 17 kb)

Table S1 (DOCX 20 kb)
