# Supplementary material for: Cultured bovine granulosa cells rapidly lose important features of their identity and functionality but partially recover under long-term culture conditions
Source: Cell Tissue Res. 2017 Feb 2;368(2):397–403. doi: 10.1007/s00441-017-2571-6 (PMC5397658; doi:10.1007/s00441-017-2571-6)
Supplement: Supplementary file 1 — (DOCX 17 kb) [file 441_2017_2571_MOESM1_ESM.docx]

**Title:** Cultured bovine granulosa cells rapidly lose important features of their identity and functionality, but partially recover under long term culture conditions

**Journal:** Cell and Tissue Research

**Authors:** Vengala Rao Yenuganti and Jens Vanselow

**Materials and Methods S1**

**Materials**

Most of the chemicals were purchased from Biochrom (Berlin, Germany) if not mentioned otherwise. Primers, FSH, IGF-1, and androstenedione were purchased from Sigma Aldrich (Steinheim, Germany). Ammonium persulfate, Blotting papers, BSA (Albumin fraction V), DMSO, Glycine, Milk powder, Methanol, NaCl, Roti-Blue (5X) and Tris were from Roth (Karlsruhe, Germany); Acrylamide/ Bis solution, collagen R, Sodium dodecyl sulfate, N,N,N′,N′-Tetramethylethylenediamine, Tween 20 from Serva (Heidelberg, Germany). The micro BCA protein assay kit, RIPA buffer, and FOXL2 antibody (Cat# PA1-31950) from Thermo Fisher Scientific (Dreieich, Germany); Immobilon-P Membrane from Millipore (Darmstadt, Germany); SOX9 (Cat# CST 82630) and Histone H3 (Cat# CST 5192) antibodies were from Cell Signaling Technology (NEB, Frankfurt/main, Germany). ECL prime, [2,4,6,7-3H] estradiol-17β from GE Healthcare, (Freiburg, Germany); Kodak Developer and fixer were purchased from Hartenstein Laborbedarf GmbH (Würzburg, Germany).

**Quantification of E2 and P4**

The levels of E2 were determined with rabbit-raised antibodies in a modified competitive 3H-RIA. The tracer, [2,4,6,7-3H]estradiol-17β, was purchased from Hartmann Analytic (Braunschweig, Germany). The lower limit for detection of E2 was 3 pg/ml and intra- and interassay coefficients of variation were 6.9 and 9.9 %, respectively. The analysis was done with 10 μl of undiluted samples in duplicates. The levels of P4 were quantified by a competitive 3H-radioimmunoassay. The tracer, [1,2,6,7-3H(N)] progesterone, was purchased from PerkinElmer (Boston, MA, USA). The minimum detection limit was 7 pg/ml and intra- and interassay coefficients of variation were 7.6 and 9.8 %, respectively. For analysis, media were diluted 1:40 in RIA-buffer and 10 μl of the dilution was measured in duplicates. The levels of radioactivity were measured in a liquid scintillation counter (TriCarb 2900 TR; PerkinElmer).
