# Supplementary material for: Cultured bovine granulosa cells rapidly lose important features of their identity and functionality but partially recover under long-term culture conditions
Source: Cell Tissue Res. 2017 Feb 2;368(2):397–403. doi: 10.1007/s00441-017-2571-6 (PMC5397658; doi:10.1007/s00441-017-2571-6)
Supplement: Supplementary file 2 — (DOCX 20 kb) [file 441_2017_2571_MOESM2_ESM.docx]

**Title:** Cultured bovine granulosa cells rapidly lose important features of their identity and functionality, but partially recover under long term culture conditions

**Journal:** Cell and Tissue Research

**Authors:** Vengala Rao Yenuganti and Jens Vanselow

**Table S1. List of primers used for real-time RT-PCR.**

| **Name** | **Sequence** | **Size (bp)** | **Accession no.** |
| --- | --- | --- | --- |
| *AR* Forward | CGACTTCTCTCCACCCGATG | 144 | NM_001244127.1**^b^** |
| *AR* Reverse | GTGGTCTCCAAACGCATGTC |  |  |
| *CCND2* Forward | CGCAGGGCCGTGCCGGACGCCAAC | 279 | NM_001076372**^a^** |
| *CCND2* Reverse | CACGGCCCCCAGCAGCTGCAGATGG |  |  |
| *CYP19A1 F*orward | GCTTTTGGAAGTGCTGAACCCAAGG | 172 | NM_174305**^a^** |
| *CYP19A1* Reverse | GGGCCCAATTCCCAGAAAGTAGCTG |  |  |
| *CDH1 F*orward | GGCTGGACCGTGAGAGTTTT | 188 | NM_001002763.1**^b^** |
| *CDH1* Reverse | GGTGATGGCCACATTAGCCT |  |  |
| *CDH2 F*orward | CCGATCCTGCAAACTGGCTA | 136 | NM_001166492.1**^b^** |
| *CDH2* Reverse | GGGATTCCATTGTCAGAGGCA |  |  |
| *ESR1 F*orward | CTGCCAAGGAGACTCGCTAC | 253 | NM_001001443.1**^b^** |
| *ESR1* Reverse | CCTCCTCTTCGGTCTTTCCG |  |  |
| *ESR2* Forward | GGTCAATCCATCCTACCCCT | 264 | NM_174051.3**^b^** |
| *ESR2* Reverse | TTCACGCCAAGGACTCTTTT |  |  |
| *FOXL2* Forward | AGCCAAGTTCCCGTTCTACG | 140 | NM_001031750.1**^b^** |
| *FOXL2* Reverse | GGTCCAGCGTCCAGTAGTTG |  |  |
| *FSHR* Forward | TCACCAAGCTTCGAGTCATCCCAAA | 189 | NM_174061**^a^** |
| *FSHR* Reverse | TCTGGAAGGCATCAGGGTCGATGTA |  |  |
| *FST* Forward | GCACTGGCCGCCTGAGCACCT | 191 | NM_175801**^b^** |
| *FST* Reverse | TGGGGCACAGACGCAGCGGG |  |  |
| *LHCGR* Forward | GCATCCACAAGCTTCCAGATGTTACGA | 205 | NM_174381**^a^** |
| *LHCGR* Reverse | GGGAAATCAGCGTTGTCCCATTGA |  |  |
| *NR5A2* Forward | ATGTGCAAGATGGCAGACCAGACG | 177 | NM_001206816.1**^b^** |
| *NR5A2* Reverse | GATGGAGCCCTCCTTTCCGTGAAC |  |  |
| *PTGS2* Forward | TACAGCACTTGAGTGGCTATCAC | 317 | NM_174445**^b^** |
| *PTGS2* Reverse | CTGGTCAATTGAAGCCTTTGATAC |  |  |
| *SOX9* Forward | ACCTGGAACTTCAGTGGCG | 147 | XM_010816647.1**^b^** |
| *SOX9* Reverse | CCAAGTAGGGGAAGGCGAAT |  |  |
| *STAR* Forward | TTGTGAGCGTACGCTGTACCAAG | 236 | NM_174189.2**^a^** |
| *STAR* Reverse | CTGCGAGAGGACCTGGTTGATG |  |  |
| *TBP* Forward | GCCTTGTGCTTACCCACCAACAGTTC | 200 | NM_001075742.1**^a^** |
| *TBP* Reverse | TGTCTTCCTGAAACCCTTCAGAATAGGG |  |  |
| *VIM* Forward | GGATGCGCTCAAAGGGACTA | 296 | NM_173969.3**^b^** |
| *VIM* Reverse | CCAGATTGGTTTCCCTCAGGT |  |  |

**a=** primers from Yenuganti et al., 2016 and **b=**primers designed from NCBI
